# Increased plasma levels of thrombopoietin in patients with severe acute pancreatitis

**DOI:** 10.1186/cc12346

**Published:** 2013-03-19

**Authors:** L Pigozzi, O Bosco, B Vizio, M Loiacono, M Lucchiari, G Mengozzi, C Moiraghi, G Montrucchio, E Lupia

**Affiliations:** 1 Città della Salute e della Scienza Hospital, Torino, Italy; 2 University of Torino, Italy

## Introduction

The aim of this study was to evaluate the accuracy of thrombopoietin (TPO) plasma levels as a biomarker of clinical severity in patients with acute pancreatitis (AP). TPO is a humoral growth factor that stimulates megakaryocyte proliferation and differentiation [1]. Furthermore, it favors platelet aggregation and polymorphonuclear leukocyte activation [2]. Elevated plasmatic concentrations of TPO have been shown in patients with critical diseases, including ACS, burn injury and sepsis [2]. In particular, clinical severity is the major determinant of elevated TPO levels in patients with sepsis [3]. AP is a relatively common disease whose diagnosis and treatment are often difficult, especially in the clinical setting of the emergency department (ED). About 20% of patients with AP develop a severe form of the disease. In order to early identify those patients affected by severe AP, several biomarkers have been studied. No data regarding TPO plasma levels in patients with AP are currently available.

## Methods

We enrolled patients with AP at the moment of the first clinical evaluation in the ED. AP patients were classified as having mild or severe forms of AP on the basis of the APACHE II score (≥8). TPO concentrations were determined by ELISA.

## Results

**Figure 1:**
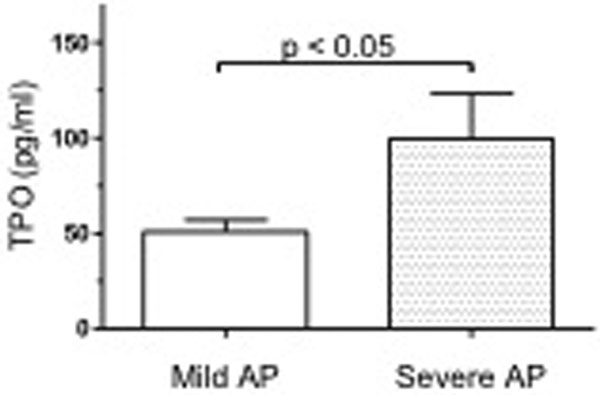
**Plasma TPO levels in patients with AP**.

We studied 41 patients with AP (17 severe and 24 mild pancreatitis). No differences for gender and age were detected between patients with mild and severe disease. TPO plasma levels were significantly higher in patients with severe AP (99.33 ± 23.68 pg/ ml) than in those with mild AP (50.81 ± 6.73 pg/ml) (Figure 1). The ROC curve led us to calculate a cutoff value of 51.55 pg/ml. This value identified correctly 12 out of the 17 patients affected by severe AP.

## Conclusion

TPO may be proposed as biomarker of clinical severity in patients with AP at the time of first evaluation in the ED. Further studies, involving larger numbers of patients, are needed in order to validate these preliminary data.
